# Domestic cattle (*Bos taurus taurus*) are motivated to obtain forage and demonstrate contrafreeloading

**DOI:** 10.1371/journal.pone.0193109

**Published:** 2018-03-07

**Authors:** Jennifer M. C. Van Os, Erin M. Mintline, Trevor J. DeVries, Cassandra B. Tucker

**Affiliations:** 1 Center for Animal Welfare, Department of Animal Science, University of California, Davis, United States of America; 2 Department of Animal Biosciences, University of Guelph, Ontario, Canada; University of Illinois, UNITED STATES

## Abstract

Domestic cattle (*Bos taurus taurus*) are adapted to digest high-roughage diets, but in confinement they are commonly fed low-roughage, high-energy diets. This practice may leave cattle with an unfulfilled need to consume forage. A way to quantify motivation is to require animals to work to access a resource. Using this method, we evaluated cattle motivation to obtain forage when fed high- or low-roughage diets during and 30 d before the study. Individual heifers were fed Sudan grass (*Sorghum × drummondii*) hay (high roughage, *n =* 6) or a diet with 12% forage (as fed, low roughage, *n* = 6) in an open feed trough. In a second trough, 200 g/d of Sudan grass hay were fed behind a push gate, to which additional weight was added daily until heifers no longer pushed. We predicted heifers would push heavier weights, show a shorter latency, and spend more time pushing the gate when fed a low- vs. high-roughage diet. Indeed, heifers fed a low-roughage diet pushed the gate immediately after hay delivery (1.7 min) and much sooner than those fed a high-roughage diet (75.7 min). On the day before they no longer pushed the gate, latency for heifers in the low-roughage treatment remained only 3.2 min after hay delivery. The suddenness with which they ceased pushing the next day suggests they were unable to move heavier weights to express their motivation. This may explain why maximum weight pushed and time spent pushing the gate did not differ between treatments. The gate pushing by heifers with unrestricted hay access is the first demonstration by cattle of contrafreeloading: performing work to obtain a resource that is simultaneously available for free. In conclusion, consuming forage is important to cattle and is affected by both their primary diet and an internal motivation to work to obtain feed.

## Introduction

Domestic cattle (*Bos taurus taurus*) are ruminants adapted to digest diets high in roughage. The repertoire of natural feeding behavior in cattle includes appetitive components related to foraging (searching for and investigating feed) and consummatory aspects such as chewing and ruminating. In extensive rangeland or pasture settings, cattle spend a large portion of the day grazing (7–13 h/d) and ruminating (5–10 h/d) [1]. In contrast, in some intensive production systems (i.e., pre-weaned dairy heifers, veal calves, and feedlots), cattle are commonly fed low-roughage, high-energy diets based on grain concentrates. For example, 48% of US feedlot cattle in the finishing phase are fed diets with >75% concentrate on a dry-matter (**DM**) basis [2]. Such diets, in theory, allow cattle to efficiently meet their nutrient requirements, but may have adverse effects on other aspects of physiology and behavior.

Long, fibrous feed particles require more chewing, consequently increasing the production of bicarbonate-rich saliva [3], which buffers rumen pH. In contrast, highly fermentable grain-based diets can cause excessive acid production in the rumen, leading to acute or subacute ruminal acidosis [4–6]. When given the opportunity, cattle experiencing ruminal acidosis make diet choices that ameliorate low rumen pH: they preferentially sort for longer feed particles [7–9] and prefer alfalfa in the form of long-stemmed hay rather than pellets [10].

Another consequence of grain-based diets being consumed at a faster rate than forage [3] is the reduced expression of natural feeding behavior. Cattle may have an intrinsic motivation to perform feeding behavior, and a diet low in roughage may not fulfill this behavioral need. Indeed, even when their rumens are filled through a fistula, dairy cows investigate the area around the empty feed trough using their tongues and noses [11]. Furthermore, when their diets are switched to maintain the same energy content but with a lower proportion of roughage, cattle increase the performance of stereotypic oral behaviors, such as tongue rolling [12], that are not observed in their normal behavioral repertoire in extensive settings [13].

An animal’s degree of motivation can be evaluated by asking it to work to gain access to a resource. In the animal motivation literature, the amount of work performed is commonly described as the price paid, drawing terminology from consumer demand in humans. Combined with measures of resource use (e.g., greater frequency and duration, along with shorter latency to approach), a willingness to pay a higher price indicates the animal values the resource more than less important items [14, 15]. The approach of evaluating motivation by asking animals to perform work has yielded valuable information across a number of species. For example, farmed mink (*Mustela vison*) will push doors weighted with nearly the equivalent of their bodyweight to gain access to a water pool [16], indicating they highly value this resource. Furthermore, when denied access to the water pool, mink showed a 33% increase in urinary cortisol [16], illustrating the interconnection between highly motivated behavior and physiological functioning.

Previous studies found that veal calves and dairy heifers fed high-energy diets were willing to perform work to obtain forage by pressing panels or pushing weights, respectively [17, 18]. However, those studies lacked a control treatment to establish the baseline level of work cattle were willing to perform when fed forage, and thus how much additional motivation they show when fed a high-energy, low-roughage diet. Working to obtain additional forage when it comprises the primary diet would be an example of contrafreeloading. To date, contrafreeloading has not been definitively observed in cattle, although it has been shown in other ruminants such as goats (*Capra hircus*) [19]. Therefore, the objective of the current study was to evaluate the motivation of feedlot cattle to obtain forage when fed a low-roughage compared to a forage-only diet. We predicted that, when presented with a weighted gate to obtain forage, cattle with ad libitum access to this feed would demonstrate contrafreeloading by pushing the gate, and that those fed a high-concentrate, low-roughage diet would push heavier weights, show a shorter latency and spend more time using the gate, and consume more forage from behind the gate.

## Materials and methods

### Subjects and housing

The study was conducted at the University of California-Davis (UC Davis), U.S.A, from September 2014 to January 2015. Twelve Angus-Hereford cross heifers (*B*. *taurus taurus*; mean ± SD age: 1.1 ± 0.1 yr) were divided into 3 sequential cohorts of 4 cattle. For at least 30 d before data collection, heifers were housed at the UC Davis feedlot in larger groups including non-study animals. Once data collection began for each cohort, heifers were housed individually for a maximum of 11 d at the UC Davis beef facility. Heifers were assigned to one of 4 pens, with treatments balanced across cohorts and pens. Each pen was 5 × 5.5 m and contained 2 adjacent 114 × 57 cm feed troughs and a metal 379-L trough with a float valve to provide water ad libitum. The pens were adjacent and were separated with livestock fencing (Powder River Inc., Provo, UT, U.S.A.). Each pen had five 1.16 × 1.74 m rubber mats (Interlock; Animat Inc., Sherbrooke, QC, Canada): 3 were placed in the center of the pen to create a lying area and the other 2 were in front of the feed troughs. The feed troughs and center aisle of the barn were covered with a solid roof. Cover was provided over the remaining areas of the pens by white tarps (Intertape Polymer Group, Montreal, QC, Canada).

### Ethics statement

The protocol (number 17826) for the study was reviewed and approved by the UC Davis Institutional Animal Care and Use Committee. All heifers received a physical exam by a veterinarian on the day they entered their individual pens and were monitored throughout the study for signs of illness or injury. We chose to house the heifers individually to quantify motivation at the individual level, because a social feeding environment would add another dimension of variability to their motivation. During the individual housing period, heifers had visual, auditory, and limited physical contact with conspecifics through the fencing separating the pens, and we observed anecdotally the performance of social grooming.

### Primary diet treatments

The treatments were the primary diets the cattle were fed. The high-roughage control treatment (*n* = 6) was Sudan grass (*Sorghum* × *drummondii*) hay, chopped to approximately 15 cm. The high-energy, low-roughage treatment (*n* = 6) was the total mixed ration typically used in the UC Davis feedlot. It contained 12% forage (as fed) and included supplements (Table 1); this approach was taken to mimic how feedlots provide this type of low-roughage feed. Sudan grass hay was chosen for the high-roughage treatment due to its uniformity and to reduce the possibility of cattle sorting for different particle sizes. The required sample size (*n* = 6 cattle per treatment) was determined with a power analysis to achieve 80% power using estimates of variability in maximum price derived from Greter et al. [17]. Assignments to the primary diets were balanced for starting bodyweight (low vs. high roughage: 320 ± 21 vs. 329 ± 27 kg, respectively, mean ± SD).

**Table 1:** Ingredients, chemical analysis, and calculated energy for the primary diets.

		Primary diet treatment	
		Low roughage	High roughage
Ingredient, %			
	Corn grain (flaked)	62.3	–
	Dried distillers grains with solubles (corn based)	15.0	–
	Tallow	2.5	–
	Molasses (cane)	6.0	–
	Alfalfa hay (early bloom)	6.0	–
	Wheat hay	6.0	–
	Sudan grass hay	–	100
	Limestone	1.12	–
	Urea	0.57	–
	Magnesium oxide	0.14	–
	Cobalt sulphate (21% Co)	0.00004	–
	Copper sulphate (25.4% Cu)	0.002	–
	Manganese sulphate	0.0095	–
	Potassium iodide (68% I)	0.000045	–
	Salt	0.3	–
	Rumensin 90^a^ (41.2 g/kg)	0.02	–
	Zinc Oxide (78% Zn)	0.0027	–
Chemical analysis			
	Dry matter (DM), %	83.6	88.8
	CP, % DM	12.6	8
	NDF, % DM	17.9	60.3
	ADF, % DM	8.6	41.9
Calculated energy			
	TDN, % DM	84.7	56.4
	NE_m_, MJ/kg	8.64	4.97
	NE_g_,MJ/kg	5.89	2.57

The primary diet treatments were a total mixed ration with 12% forage (low roughage) or 100% Sudan grass hay (high roughage). Ingredient composition is listed on an as-fed basis. Chemical analysis and energy calculations were performed by Cumberland Valley Analytical Services, Hagerstown, MD, U.S.A. using the formulas of Weiss [20] and NRC [21].

^a^Elanco Animal Health, Greenfield, IN, U.S.A.

Researchers previously found that heifers with more experience (34 vs. 8 d) with a high-concentrate, low-roughage diet show greater sorting for longer feed particles [7]. Therefore, to minimize possible changes in feeding behavior during data collection, heifers were fed their assigned treatment diet for at least 30 d beforehand while group-housed at the UC Davis feedlot. No data were collected during this period, but as a result of the differing nutritive quality of these diets, average bodyweight differed between treatments at the end of the experiment (low vs. high roughage: 403 ± 19 vs. 356 ± 21 kg, respectively, mean ± SD).

### Push gate design

**Fig 1:**
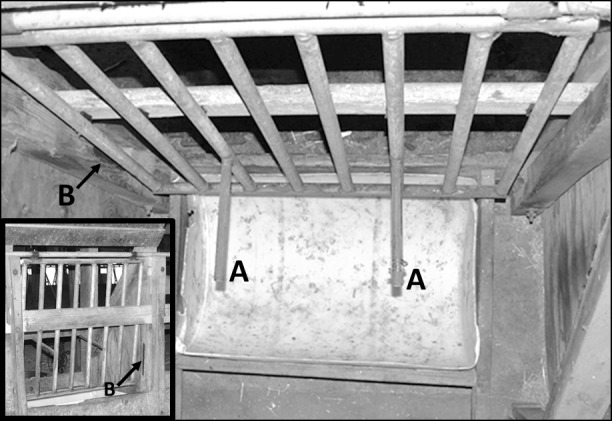
Trough with weighted gate heifers had to push to access hay. The main photo is an overhead view of the outside of the gated trough, and the inset shows the gate from inside the pen. The gate had 2 bars (A) to which weight plates were added. A black line (B) was painted on the wall to mark the position of the gate when fully closed.

To test motivation, a 27-kg gate was mounted inside each pen in front of the right-hand feed trough such that a heifer had to push the gate and hold it open with her head to access the feed (gated trough; Fig 1; see S1 Video for an example of gate use). Each gate was 101 × 95 cm and was constructed with 3-cm-diameter metal pipes spaced 11 cm apart to allow the heifers to see into the feed trough. Attached perpendicularly to the outside of each gate were two 2.5-cm-diameter metal bars to which weight plates could be added.

### Training, testing, and feeding procedures

Heifers were fed twice daily at 0800 and 1400 h (60 and 40% of the primary ration, respectively). On the day the heifers entered their individual pens, the ration for the primary diet was calculated to 4% of bodyweight. Thereafter, to ensure the primary ration was available ad libitum, feed refusals were weighed daily before the morning feeding and rations were adjusted on an individual basis to 115% of the amount consumed in the previous 24 h.

#### Training procedure

Heifers were trained to use the gated trough in a stepwise manner beginning on the day they entered their pens. A heifer advanced to the next step once she pushed the gate 3 times (for at least 2 s each time) in 24 h. In step 1, 70% of the heifer’s primary ration was placed in the open trough and the remaining 30% was placed in the gated trough. The gate was tied open to leave a 25-cm gap. In step 2, the gate was closed and the primary ration was divided into the 2 troughs as in step 1. In the third and final step, 100% of the primary ration was placed in the open trough. The gate was again tied open to leave a 25-cm gap with 200 g of Sudan grass hay placed behind it. After 24 h, the gate was closed, completing the training phase. All heifers completed training within 4 d. None spent more than 24 h at any step with the exception of 1 heifer that did not feed from either trough for the first 18 h in the pen and thus spent 42 h in step 1.

#### Testing procedure

During testing, 200 g of Sudan grass hay (177.6 ± 5.9 g, mean ± SD, on a DM basis) was placed behind the closed weighted gate at the morning feeding, when the primary diet was simultaneously fed in the open trough. The hay was offered in this small amount to minimize the likelihood of causing cumulative changes in rumen pH or fill. If the heifers used the gate, 34 kg of additional weight was added to it at the next morning feeding. This amount was chosen based on pilot tests, which showed that increasing by lower weight increments (i.e., 11 kg) presented an insufficient additional challenge for the heifers. If a heifer failed to use the gate during a 24-h period, testing was concluded. The final weight each heifer pushed (including the 27-kg gate) on the previous day (d 0) was converted to percent of bodyweight, and this value was designated the maximum price.

#### Behavioral measures

Each pen was recorded continuously 24 h/d with 2 digital video cameras (WV-BP334 black-and-white CCTV video cameras; Panasonic Corp. of North America, Secaucus, NJ, U.S.A.), which were mounted 2.65 m above each feed trough. All cameras had adjustable lenses (13VS2812ASII; Tamron, Commack, NY, U.S.A.), were set to record at medium quality and 15 frames/s, and were connected to a digital video recorder with GeoVision Surveillance System software (version 8.4; GeoVision Inc., Taipei, Taiwan). Red lights were placed over each pen for night-time visibility.

To quantify use of the gated and open feed troughs, 9 observers scored the video recordings continuously for the start and end times to the nearest second. Use of the open trough began when a heifer’s nose and chin crossed into the feed trough for at least 2 s and ended when she removed these parts of her head from the feed trough for at least 2 s. For the gated trough, a black line was painted on the wall marking the position of the gate when fully closed (Fig 1). Use of the gated trough began when the gate moved away from the painted line for at least 2 s and ended when the gate returned to the marked line. Reliability was determined using the coefficient of concordance [22, 23]. Interobserver reliability for pairs of observers (each compared against the trainer, E. M. Mintline) ranged from κ = 0.76 to 0.93 and κ = 0.81 to 0.95 for the start and end times, respectively, of open trough use and κ = 0.97 to 0.98 for both the start and end times of gated trough use. Intraobserver reliability ranged from κ = 0.69 to 0.96 and κ = 0.75 to 1.0 for the start and end times, respectively, of open trough use and κ = 0.90 to 1.0 and κ = 0.93 to 1.0 for the start and end times, respectively, of gated trough use. These measures of reliability indicated substantial (κ = 0.61 to 0.80) to almost-perfect (κ > 0.80) agreement, as suggested by Landis and Koch [24].

### Statistical analysis

Because maximum price differed for individual heifers, so did the number of days they took to reach this point (range: 4 to 5 vs. 5 to 7 d in the high- and low-roughage treatments, respectively). Therefore, to allow for consistent comparisons among heifers, their responses for all measures excluding maximum price were standardized relative to the final day each heifer pushed the gate (d 0), and only 5 d (d –4 to 0) were included in analysis.

#### Excluded data

To reduce the possibility that feeding from the gated trough could occur due to insufficient amounts of feed being provided in the open trough, data between d –4 to 0 for all response variables were excluded for days on which the refusals from the open trough were ≤0.3 kg. Days with excluded data were d –4 and 0 in the high-roughage treatment (final *n* = 5) and d –1 in the low-roughage treatment (final *n* = 4).

#### Statistical models

All analysis was conducted using mixed models (PROC MIXED) in SAS software version 9.4 [25]. The residuals for each model were evaluated for homoscedasticity and normality. Treatment differences in maximum price were compared using a model with a fixed term for treatment. The other dependent variables were recorded daily: the total time heifers spent using each feed trough, their latency to use them after the morning feed delivery, and dry matter intake (**DMI**). Intake rate (g of DM/min) was calculated by dividing DMI by the total time heifers used each trough. Differences between the primary diet treatments were analysed for each response using models with fixed terms for treatment, day (as a continuous variable), and the treatment × day interaction. All models included a random term for heifer nested within treatment and used the containment method to estimate degrees of freedom. A variance-components covariance structure was selected based on the lowest Aikaike information criterion value. The rate of DMI from the gated trough and the latency to use both feed troughs were not normally distributed, as determined by the Shapiro-Wilk test statistic and by visual inspection of the residuals. In addition, these variables appeared to show potentially non-linear patterns across days. Therefore, a Box-Cox analysis (PROC TRANSREG, with 0.01 added to all values to account for zeroes) was used to determine the appropriate transformations. The fourth root was used to transform the latency to use and the rate of DMI from the gated trough, and the natural log was used to transform the latency to use the open trough. A significance level of *P* < 0.05 (2-tailed) was used. Finally, descriptive data for mean ± SEM trough use by hour of the day were generated using PROC SUMMARY.

## Results

### Overall treatment effects

**Fig 2:**
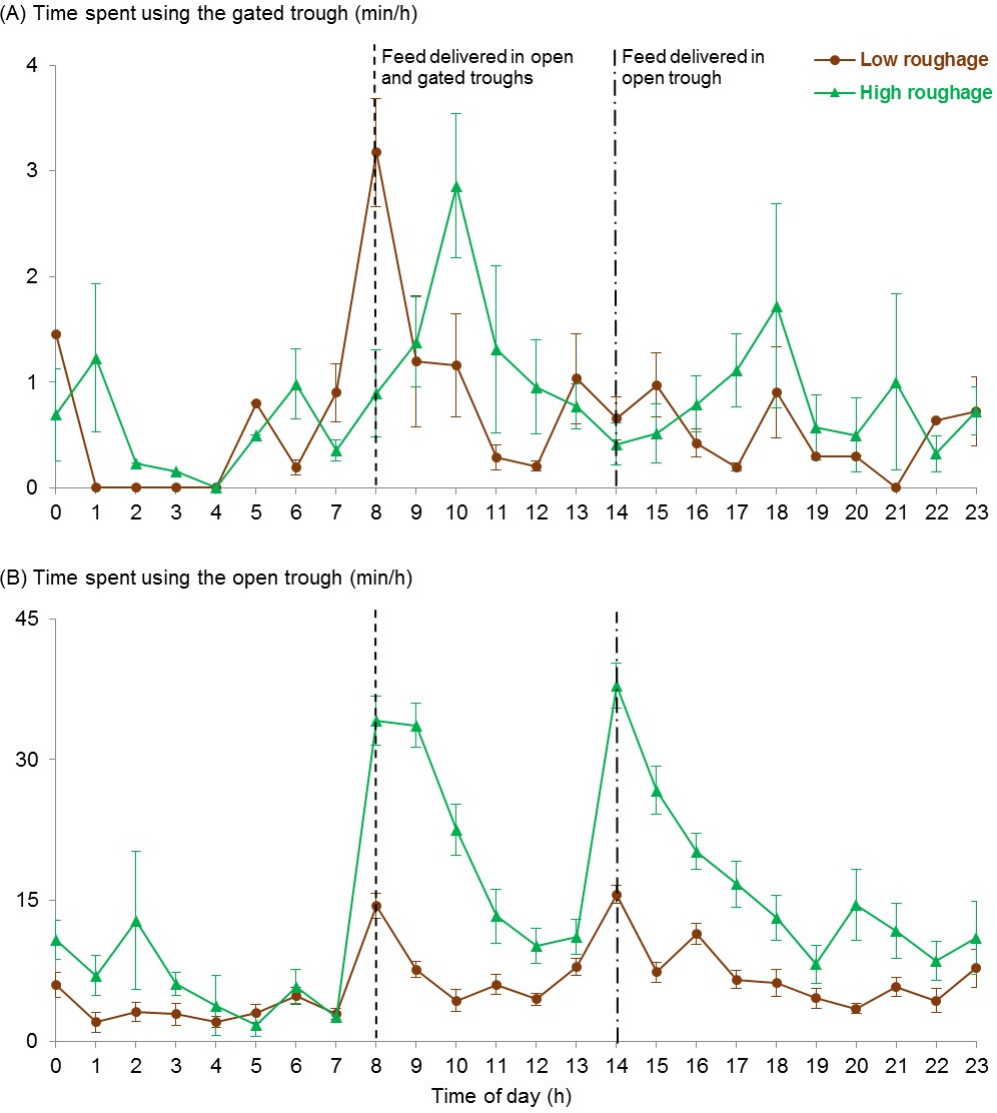
Descriptive data for diurnal patterns of trough use. The mean ± SEM amounts of time heifers spent using the (A) gated or (B) open feed trough are shown by hour of the day. At 0800 h, all heifers were fed Sudan grass hay behind a weighted gate that must be pushed open to gain access. The primary diet treatments were delivered to the open trough at 0800 and 1400 h, and consisted of either a total mixed ration with 12% forage (as fed, low roughage, *n =* 6) or 100% Sudan grass hay (high roughage, *n =* 6).

Descriptive data for diurnal patterns of gated and open trough use are shown in Fig 2A and 2B, respectively. Following hay delivery, heifers fed a low-roughage diet pushed the gate immediately and did so sooner than those fed a high-roughage diet [back-transformed means: 1.7 vs. 75.7 min, respectively; 95% confidence intervals (**CI**): 0.2–6.4 vs. 40.0–131.0 min, respectively; F_1,10_ = 34.7, *P* < 0.001]. This pattern was reversed for the open trough: at the morning feeding, those in the high-roughage treatment began to consume their primary diet sooner than those in the low-roughage treatment (back-transformed means: 0.1 vs. 0.9 min, respectively; CI: 0–0.1 vs. 0.4–2.1 min, respectively; F_1,10_ = 12.0, *P* = 0.006). Furthermore, heifers in the low-roughage treatment used the gated trough before the open trough 57% of the time, whereas those in the high-roughage treatment did so in only 8% of observations.

Regardless of the primary diet, heifers in the low- and high-roughage treatments showed similar overall use of the gate (4.9 vs. 5.8 min/d, respectively, SEM: 0.6 min/d in both treatments; 12.6 vs. 11.9 pushes/d, SEM: 1.2 vs. 1.1 pushes/d), hay intake (83 vs. 70% of the amount fed, SEM: 5% in both treatments), and intake rates from this trough (back-transformed means: 60 vs. 48 g of DM/min; CI: 25–124 vs. 20–98 g of DM/min, respectively; F_1,10_ ≤ 1.6, *P* ≥ 0.24). The heifers in the low-roughage treatment consumed their primary diet at a faster rate than those in the high-roughage treatment (73 vs. 19 g of DM/min, respectively, SEM: 3.5 vs. 0.7 g/min), resulting in less time spent using the open trough (1.6 vs. 4.7 h/d, SEM: 0.3 h/d in both treatments; F_1,10_ ≥ 50.4, *P* < 0.001). Heifers in the low- and high-roughage treatments were willing to pay similar maximum prices to access hay (46 vs. 44% of bodyweight, respectively, SEM: 3% in both treatments; *F*_1,9_ = 0.1, *P* = 0.73; Table 2).

**Table 2:** Bodyweight and maximum weight of a gate heifers pushed to access hay.

		Maximum weight pushed		
Primary diet treatment	Final bodyweight, kg	kg	% of bodyweight^a^	days^b^
Low roughage				
	367	232	63	7
	404	163	41	5
	[405]	[197]	[49]	[6]
	407	197	49	6
	413	163	40	5
	422	163	39	5
High roughage				
	330	163	50	5
	341	163	48	5
	349	163	47	5
	359	129	36	4
	373	163	44	5
	387	163	42	5

For 30 d before the start of data collection and throughout the study, heifers had ad libitum access to primary diet treatments of either total mixed ration with 12% forage (as fed, low roughage, *n =* 6) or 100% Sudan grass hay (high roughage, *n =* 6).

^a^Treatments were compared based on the percentage of final bodyweight heifers pushed. Data for 1 heifer in the low-roughage treatment, identified in brackets, were excluded from final comparisons for this measure.

^b^The number of days each heifer pushed the gate is indicated. Weight was added to the 27-kg gate daily in 34-kg increments.

### Patterns across days

**Fig 3:**
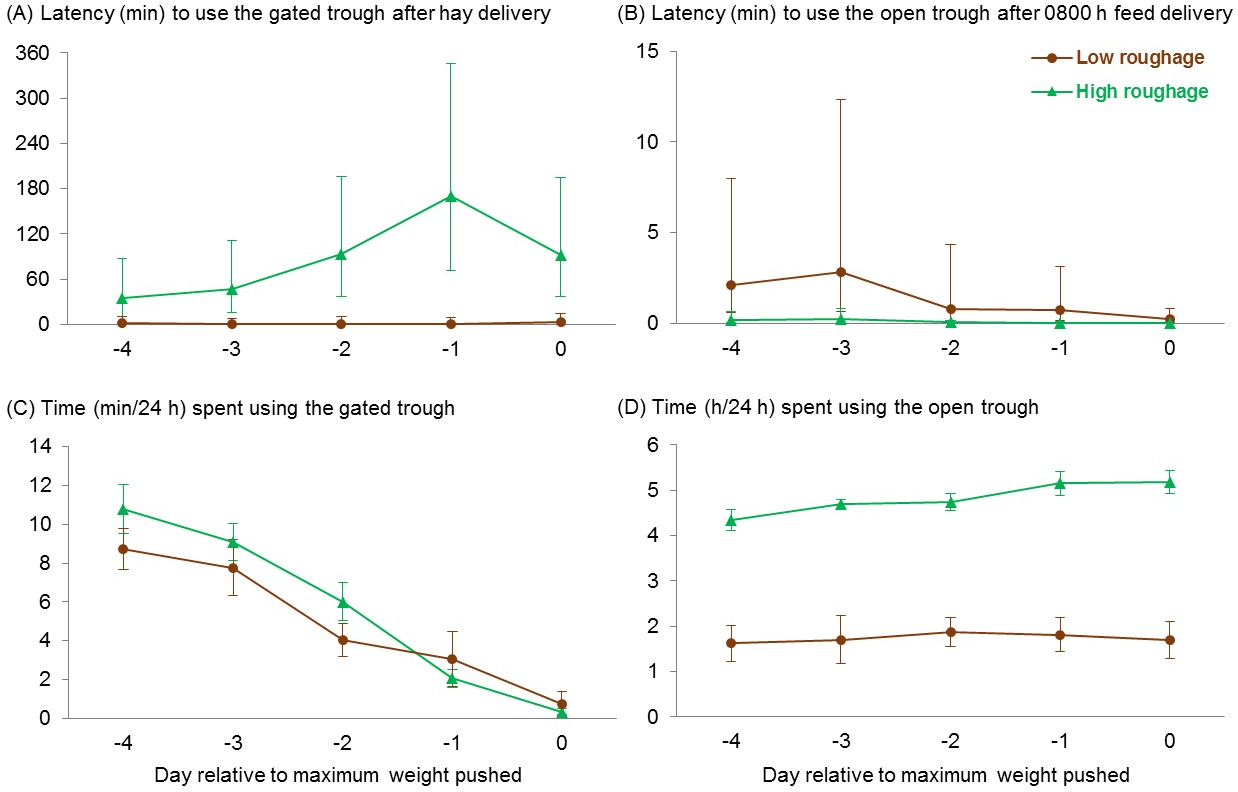
Latency and time spent using each trough. Back-transformed means and 95% confidence intervals are shown for the latency to use the (A) gated and (B) open trough after the 0800 h feed delivery and means ± SEM for the total time heifers spent using the (C) gated and (D) open trough. All heifers were fed 200 g/d of Sudan grass hay behind a gate that must be pushed to gain access, and to which additional weight was added daily until cattle no longer used it. In the unrestricted open trough, heifers had free access to either a total mixed ration with 12% forage (as fed, low roughage, *n =* 6) or 100% Sudan grass hay (high roughage, *n =* 6). To allow for consistent comparisons among heifers, behavior was evaluated on 5 d (relative to the day they pushed the maximum weight), and data are presented across days. Day was included in the model as a continuous variable, but was summarized as categorical for graphing purposes.

**Fig 4:**
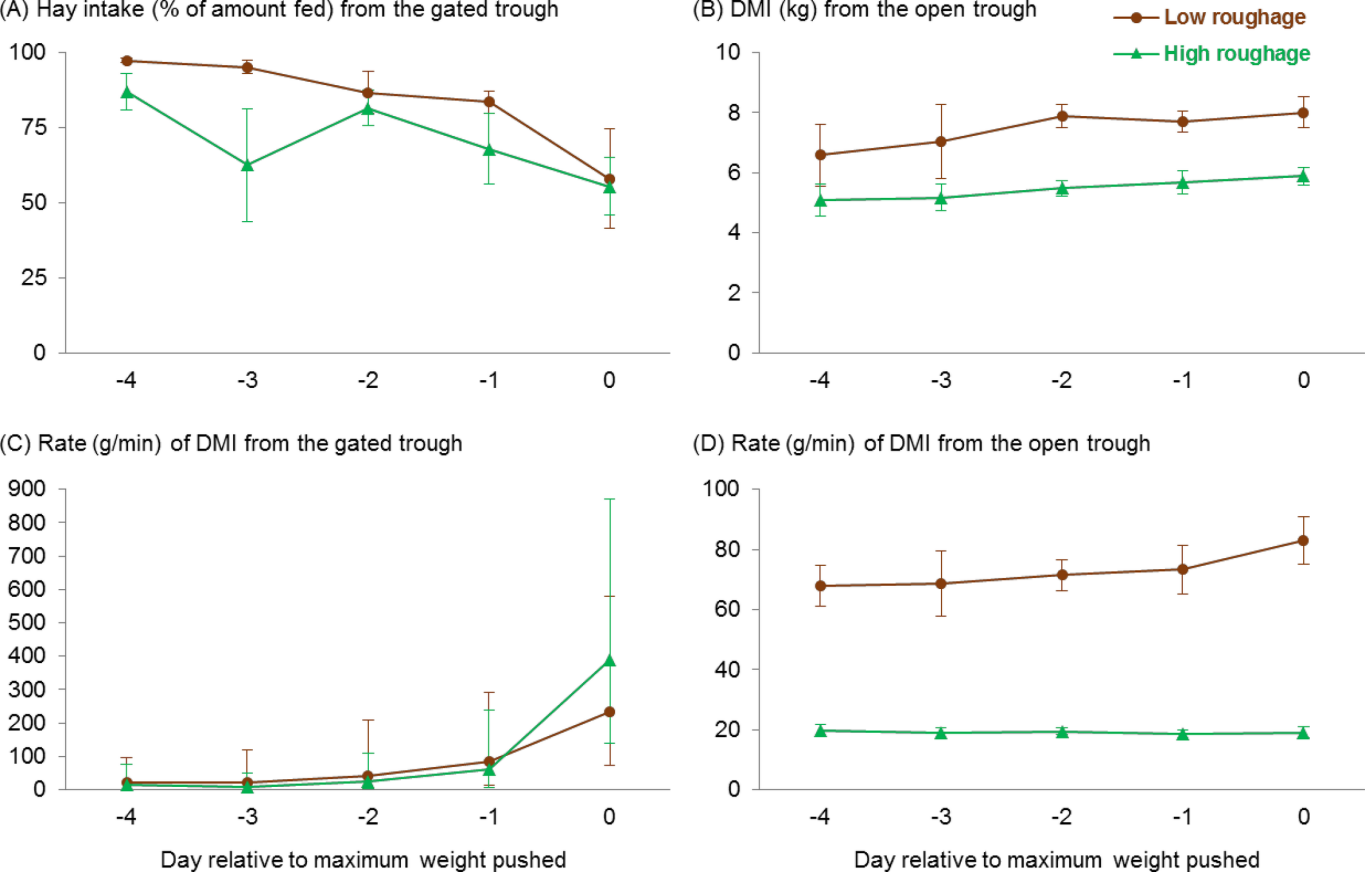
Feed intake and feeding rate from each trough. Means ± SEM are shown for intake of (A) Sudan grass hay from the gated trough and (B) the primary diet treatments from the open trough, (C) back-transformed means and 95% confidence intervals for the feeding rate from the gated trough, and (D) means ± SEM for the feeding rate from the open trough. All heifers were fed 200 g/d of Sudan grass hay behind a gate that must be pushed to gain access, and to which additional weight was added daily until cattle no longer used it. In the unrestricted open trough, heifers had free access to either a total mixed ration with 12% forage (as fed, low roughage, *n =* 6) or 100% Sudan grass hay (high roughage, *n =* 6). To allow for consistent comparisons among heifers, behavior was evaluated on 5 d (relative to the day they pushed the maximum weight), and data are presented across days. Day was included in the model as a continuous variable but was summarized as categorical for graphing purposes.

As the weight on the gate increased, heifers took longer to start pushing it (Fig 3A) and used the open trough sooner after the morning feed delivery (Fig 3B; day: F_1,31_ ≥ 4.4, *P* ≤ 0.045), regardless of their primary diet (no treatment × day interactions, F_1,31_ ≤ 2.6, *P* ≥ 0.12). In addition, as the weight on the gate increased, heifers spent less time using it (Fig 3C; day: F_1,31_ = 189.8 *P* < 0.001), and this decrease over time was more marked for those in the high-roughage treatment (treatment × day interaction: F_1,31_ = 5.3, *P* = 0.029). As the gate became heavier, heifers in both treatments pushed the gate less frequently and ate less hay from the gated trough (Fig 4A), but they compensated by consuming it at a faster rate (Fig 4C; day: F_1, 31 to 33_ ≥ 24.2, *P* < 0.001; no treatment × day interactions: F_1,31_ ≤ 2.0, *P* ≥ 0.16). Heifers increased the amount of time they spent using the open trough across days (Fig 3D; day: F_1,31_ = 6.0, *P* = 0.020) while consuming their primary rations at a consistent rate (Fig 4D; no effect of day: F_1,31_ = 0.3, *P* = 0.57), resulting in increased intake over time (Fig 4B; day: F_1,33_ = 9.6, *P* = 0.004; no treatment × day interactions, F_1, 31 to 33_ ≤ 2.3, *P* ≥ 0.14).

## Discussion

This study provides evidence that confined cattle are motivated to obtain supplemental forage, particularly when fed a high-energy diet with only 12% forage (as fed). Immediately after feed delivery, individually housed cattle fed a low-roughage diet pushed the gate to obtain hay before consuming their primary diet the majority of the time, and they worked to obtain forage much sooner than those fed a high-roughage diet. Contrary to our predictions for the other measures of motivation, however, heifers in both treatments pushed similar maximum weights and showed comparable gate use and hay consumption from this trough. The considerable gate use by heifers in the high-roughage treatment is the first demonstration by cattle of contrafreeloading, wherein animals expend effort to obtain a resource even when it is otherwise freely available [26].

### Motivation to obtain forage when fed a low-roughage diet

The results for latency to push the gate supported our hypothesis that cattle fed a high-energy, low-roughage diet would be more motivated to obtain forage compared to those fed a high-roughage diet. When hay was delivered behind a weighted gate, the heifers fed a low-roughage diet immediately pushed it, and thus their peak use occurred during the first hour after the morning feeding. In contrast, those fed a high-roughage diet took over an hour longer to begin using the gate, with their peak use occurring in the second hour after hay delivery. Nonetheless, it was surprising that those fed a low-roughage diet used the gate so soon, and that they did so before consuming their primary diet the majority of the time. Rumen pH decreases in the hours following a meal, particularly when high in fermentable carbohydrates [27], and domestic sheep (*Ovus aries*) select higher- or lower-roughage diets in accordance with changes in the rumen environment during the course of a day [28]. In our study, the short latency heifers showed to approach the gate could, in part, be explained by consuming the high-energy, low-roughage diet for 30 d before data collection. Indeed, more experience (34 vs. 8 d) with this type of diet results in greater sorting for longer, fibrous particles [7]. These responses perhaps reflect a more persistent change in internal state [29], and future work could examine whether the latency to obtain forage depends on the amount of experience with a high-energy, low-roughage diet, as well as how this relates to patterns of rumen pH within and across days. Alternatively, the immediate gate use in the current study could reflect a motivation to obtain dietary fiber based on a need to express natural feeding behavior.

The optimal diet model predicts that animals should make decisions to maximize their energy intake [30]. Indeed, when heifers are offered feed components separately, they sometimes choose to consume a high proportion of their diet as high-energy concentrate (e.g., 90–92% on a DM basis) [31]. In contrast, the results from the current study support the idea that, under some circumstances, ruminants make foraging trade-offs to maximize long-term survival rather than immediate energy intake [30, 32]. For example, extensive previous work has demonstrated that grazing cattle avoid plant defenses such as toxins [33]. More recently, a growing body of evidence suggests that confined cattle also change their feeding behavior to maintain the rumen environment by consuming roughage. Dairy heifers fed a low-roughage diet had a shorter latency to push a gate and were willing to push heavier weights to obtain oat straw, when it was offered several hours after a meal [17], compared to those fed a high-roughage diet. Veal calves fed high-energy diets were likewise willing to work to access forage by pressing panels [18]. In addition, longer particles may increase both chewing and rumen pH, which may explain why dairy cows experiencing ruminal acidosis altered their preferences for the form of their alfalfa diet in favor of long-stemmed hay over pellets [10]. Likewise, both beef and dairy cattle preferentially sorted for longer feed particles [7–9] when suffering from acute bouts of ruminal acidosis, as did dairy calves when they were offered a total mixed ration in addition to their primary diet of high-energy calf starter [34]. In future studies of ruminant motivation to obtain roughage, it would be informative to quantify feed particle size.

In contrast with our predictions, the other measures of motivation (maximum price, frequency and time spent using the gate, and hay intake from the gated trough) were similar regardless of whether heifers were fed a low- or high-roughage diet. The method of using weight to impose a price has limitations, as bodyweight can affect pushing ability, and this variable was confounded with treatment. To account for this, we expressed maximum price as a percent of bodyweight, but this approach may have masked treatment differences. In addition, maximum price may not have reflected the extent of the heifers’ motivation due to a ceiling effect on their ability to continue pushing heavier weights. Indeed, on the final day they pushed the gate, heifers in the low-roughage treatment approached the hay 3.2 min after its delivery. The suddenness with which they failed to push open the gate the next day suggests they were no longer able, rather than no longer willing, to do so. Failed attempts to open the gate could be recorded in future studies using load cells affixed to the gate or by designing the gate such that a heifer’s intention to open it was clear and could be consistently scored from video. In further support of the idea that heifers remained motivated as the weight increased, they compensated by consuming the hay at a faster rate. This finding is consistent with the increased rate of intake dairy cows showed when a time constraint was imposed on feeding [35]. However, our calculation of intake rate assumed that heifers obtained and ingested hay while actively pushing the gate, which may have affected our estimate of this measure.

The degree of effort expended by heifers in our study was surprisingly high: maximum price ranged from 36–63% of bodyweight compared to only <10% of bodyweight in Greter et al. [17]. The high values in our study suggest that those in the high-roughage treatment perceived some benefit to pushing the gate, and we discuss possible explanations below. Further research is needed to establish a gold standard for the upper limit of the weight heifers would be willing or able to push, for example to obtain feed following deprivation. Different methods of assessing motivation could also be compared, such as requiring cattle to walk various distances [36], by creating trade-offs between resources [37], or by adding an aversive component to the gate to increase the cost of pushing it, as we did in a recent follow-up study [38].

Cattle use of the open trough was consistent with previous research. Delivery of fresh feed stimulates feeding behavior in confined cattle [39], and heifers in both treatments showed greatest use of the open trough during the hour following each delivery of their primary diet. The low-roughage diet was consumed 3 times faster than the high-roughage diet, consistent with other studies [3, 17], resulting in shorter feeding time. Intake of the high-energy diet was, on average, 1.6 to 2.0% of bodyweight, which is within the range (1.4–2.4% of bodyweight) consumed by feedlot cattle in other studies [40–42].

### Contrafreeloading in the high-roughage treatment

Heifers in the high-roughage treatment demonstrated contrafreeloading, a phenomenon in which animals expend effort to obtain a resource even when it is simultaneously available freely and in abundance [26]. Contrafreeloading has been observed across several species, not only by rats (*Rattus norvegicus*) [43], chickens (*Gallus gallus*) [44], and swine (*Sus scrofa*) [45] to obtain feed, but also by goats (*C*. *hircus*) for drinking water [19] and by macaques (*Macaca fuscata*) to watch video clips [46]. Our experiment provides the first evidence of contrafreeloading in cattle. Other researchers claimed that grazing cattle contrafreeloaded, but this was based only on more active foraging behavior shown by heritage relative to commercial breeds [47, 48]. Another group suggested contrafreeloading was observed in confined dairy cows, but the free feed was of a different type and was not provided simultaneously [49]. Finally, calves in another study obtained Lucerne hay by pressing one panel more than another that required fewer presses, but this type of feed was never freely offered, and thus their behavior was termed ‘contracheaploading’ [18].

The contrafreeloading we observed is consistent with the idea that, under some circumstances, cattle expend energy while foraging to obtain benefits besides maximizing immediate caloric intake. In addition to maintaining rumen health, their feeding choices may be influenced by other ultimate or proximate factors. An ultimate explanation for contrafreeloading could be gathering information about available food sources, whereas proximate factors include hunger, performing species-typical foraging behavior, and expressing control over the environment. We discuss each potential explanation in turn.

A functional hypothesis for contrafreeloading is that gathering information about potential food sources is adaptive for long-term survival, particularly in uncertain environments [26, 50]. Evidence supporting this hypothesis has been reported for several species, including gerbils (*Meriones unguiculatus*) [51], starlings (*Sturnus vulgaris*) [52], and chickens (*G*. *gallus*) [44]. Our study environment, however, had little uncertainty: in the high-roughage treatment, the diet in the gated trough was the same during training and testing, and the timing and amount fed was consistent across testing days. Furthermore, cattle could see into the trough behind the gates, and animals are predicted to expend less effort to gather information when food sources can be visually inspected [51, 52]. Therefore, contrafreeloading in our study may be better explained by proximate benefits of pushing the gate.

A potential proximate explanation is that cattle used the gated trough due to hunger. However, this is unlikely, as the primary diets were fed ad libitum and hay would be predicted to promote rumen fill and satiety [53, 54]. Rather, pushing the gate may have been inherently rewarding if this allowed heifers to express species-typical foraging behavior [26]. Cattle may be motivated to perform a certain amount of foraging behavior regardless of physiological satiation. Indeed, when their feeding durations are limited experimentally, confined dairy cows use their tongues and noses to investigate the area around the empty trough despite having their rumens filled through a fistula [11]. Likewise, when changed to a lower-roughage diet, cattle increase the performance of abnormal oral behaviors, despite receiving equivalent energy content [12]. In our study, heifers in the high-roughage treatment spent 31–64% less time feeding compared with grazing cattle [1], and pushing the gate perhaps represented investigative foraging behavior. However, other factors also likely influence gate use, given that heifers in the low-roughage treatment spent less time feeding overall than those in the high-roughage treatment, but did not show more gate use.

Another proximate explanation for contrafreeloading is that, in the predictable setting of our study, cattle may have perceived pushing the gate as a form of environmental enrichment, given that the housing was relatively barren and the heifers had only limited social contact. Using the gate may have been rewarding if this alleviated boredom, created a sense of control over the environment, or allowed heifers to express agency by handling a challenge [55]. Controlling the environment or expressing agency were suggested to explain why contrafreeloading was shown by Japanese macaques to watch movie clips [46] and by human children to obtain marbles [56] or pennies [57]. Perceived control or agency may also explain why dairy heifers who performed an operant task to access feed showed greater excitement than those rewarded without expending effort, as measured by heart rate and locomotor patterns such as jumping, kicking, or bucking [58]. To evaluate why pushing the gate may be rewarding for cattle, future studies could add comparisons to non-ingestible rewards or an empty trough, manipulate opportunities to perform investigative foraging behavior, as well as examine behavioral and physiological measures of excitement.

## Conclusions

Individually indoor-housed cattle are motivated to obtain supplemental forage, particularly when fed a low-roughage, high-concentrate diet. Whereas cattle fed a high-roughage diet did not use the gate until over an hour after feed delivery, those fed a low-roughage diet worked to obtain hay immediately after its delivery and did so before consuming any of their primary diet the majority of the time. Regardless of their primary diet, cattle pushed nearly half of their bodyweight to access a small portion of hay. The use of the gate by heifers with free access to hay is the first demonstration of contrafreeloading in cattle. In conclusion, consuming roughage is important to domestic cattle, and this desire is affected by both their primary diet and an internal motivation to work to obtain feed.

## Supporting information

S1 Video Example of contrafreeloading and gate use. A heifer pushes a 27-kg gate with her head to obtain an additional portion of Sudan grass hay, her primary diet that is simultaneously freely available in an adjacent open trough.
(MP4)

S1 Appendix Summarized data used for statistical analysis. The data used for statistical analysis are given for heifers in the high- (*n* = 6) and low-roughage (*n* = 6) primary diet treatments. These include latency to use the gated and open troughs after the morning feeding, total time spent using each trough, and total feed intake and rate from each trough on d –4 to 0 relative to the final day the heifers pushed the gate, along with maximum price (the final weight, including the gate, they pushed). Data points excluded from analysis are indicated.
(XLSX)
